# Effects of Nebivolol on Endothelial Gene Expression during Oxidative Stress in Human Umbilical Vein Endothelial Cells

**DOI:** 10.1155/2008/367590

**Published:** 2008-04-17

**Authors:** Ulisse Garbin, Anna Fratta Pasini, Chiara Stranieri, Stefania Manfro, Chiara Mozzini, Veronica Boccioletti, Andrea Pasini, Mattia Cominacini, Stefano Evangelista, Luciano Cominacini

**Affiliations:** ^1^Internal Medicine D, Department of Biomedical and Surgical Sciences, University of Verona, 37126 Verona, Italy; ^2^Preclinical Development Department, Menarini Ricerche Spa, Via Sette Santi 1, 50131 Firenze, Italy

## Abstract

The endothelium plays a key role in the development of atherogenesis and its inflammatory and proliferative status influences the progression of atherosclerosis. The aim of this study is to compare the effects of two beta blockers such as nebivolol and atenolol on gene expression in human umbilical vein endothelial cells (HUVECs) following an oxidant stimulus. HUVECs were incubated with nebivolol or atenolol (10 micromol/L) for 24 hours and oxidative stress was induced by the addition of oxidized (ox)-LDL. Ox-LDL upregulated adhesion molecules (ICAM-1, ICAM-2, ICAM-3, E-selectin, and P-selectin); proteins linked to inflammation (IL-6 and TNFalpha), thrombotic state (tissue factor, PAI-1 and uPA), hypertension such as endothelin-1 (ET-1), and vascular remodeling such as metalloproteinases (MMP-2, MMP-9) and protease inhibitor (TIMP-1). The exposure of HUVECs to nebivolol, but not to atenolol, reduced these genes upregulated by oxidative stress both in terms of protein and RNA expression. The known antioxidant properties of the third generation beta blocker nebivolol seem to account to the observed differences seen when compared to atenolol and support the specific potential protective role of this beta blocker on the expression of a number of genes involved in the initiation and progression of atherosclerosis.

## 1. INTRODUCTION

Nebivolol, a third-generation beta-blocker endowed with high selectivity for *β*_1_ adrenoceptors [1] and with the capability to release nitric oxide (NO) from endothelium [2], has been shown to significantly affect the amount of reactive oxygen species (ROS) released from human endothelial cells under oxidative stress [3–6]. It seems that this effect is produced, at least in part, by the inhibition of endothelial NADPH oxidase [7], a key enzyme in the ROS formation inside the eucariotic cells [8], and also by the direct ROS scavenging effect of the drug [3, 4, 6, 9].

The oxidative status of cells of the cardiovascular system is dependent on the balance between ROS and endogenous antioxidants and recent studies indicate that increase in ROS is the intracellular signal and marker of their condition [10, 11]. Extracellular factors such as hypercholesterolemia with increased oxidized low-density lipoprotein (ox-LDL), hyperglycemia with enhanced production of advanced glycation end products, hypertension with high levels of angiotensin II, endothelins, and cytokines can move the balance toward the prooxidant state of the cell with a change of its redox state and enhanced formation of ROS [10, 11]. The change of redox form of the cell induces the activation of a series of proteins and intracellular enzymes (epidermal growth factor receptor, c-Src, p38 mitogen-activated protein kinase, Ras, Akt/protein kinase B) as well as transcription factors redox-sensitive (NF-*κ*B, AP-1) and the consequent induction of genes involved in the endothelial functions (adhesion molecules, prothrombotic factors) and in the change of the extracellular matrix that is involved in the formation and progression of the atherosclerotic disease.

In view of the previous observation of the nebivolol noticeable inhibitory activity on ROS formation [3–6], the aim of the present work is to evaluate the potential effect of the drug on the redox-sensitive genes involved in the atherosclerotic plaque formation.

## 2. MATERIALS AND METHODS

### 2.1. Cell culture and experimental design

Human umbilical vein endothelial cells (HUVECs) were obtained according to the method of Jaffe et al. [12] and used at used from passage 2 to 4. The cells were grown in 75 cm^2^culture flasks (Falcon, Becton Dickinson, Lincoln Park, NJ, USA) filled with 10 mL of M-199 (Sigma-Aldrich, St. Louis, Mo, USA) containing 10% fetal calf serum (Seromed, Berlin, Germany), 2 mM glutamine (Seromed, Berlin, Germany), 30 *μ*g ml^−1^ endothelial cell growth supplement (Sigma-Aldrich, St. Louis, Mo, USA), 100 *μ*g ml^−1^ heparin (Sigma-Aldrich, St. Louis, Mo, USA), 100 U mL^−1^ penicillin-streptomycin (Sigma-Aldrich, St. Louis, Mo, USA), 100 *μ*g mL^−1^ streptomycin (Sigma-Aldrich, St. Louis, Mo, USA), and 2.5 *μ*g mL^−1^ amphotericin (Sigma-Aldrich, St. Louis, Mo, USA). The flasks were incubated at 37°C, 100% humidity, and 5% of CO_2_. The medium was refreshed every 2 days. At the beginning of each experiment, the cells were harvested by trypsinization, using 0.05% trypsin (Sigma-Aldrich, St. Louis, Mo, USA) and 0.537 mM ethylenediamine tetraacetic acid (EDTA) in phosphate buffered saline without calcium and magnesium (Seromed, Berlin, Germany). The trypsin was inactivated by dilution, and the cells were washed and counted. Cells were plated at a concentration of 40000 cells ^2^−1^^ on a multiwell plate (9.6 cm^2^ well^−1^) (Falcon, Becton Dickinson, Lincoln Park, NJ, USA), have grown for 2 days, and then used for the incubations.

HUVECs were harvested and characterized as to acetylated LDL binding and factor VIII expression, according to established and previously described techniques [13]. To assess cell survival, hexosaminidase, a stable cytosolic enzyme released by cells when they undergo lysis, was measured according to the method of Landegren [14].

Dl-nebivolol (Berlin Chemie, Menarini Group, Firenze, Italy) and atenolol (Sigma-Aldrich, St. Louis, Mo, USA) were dissolved in ethanol and then diluted at the final concentration in culture medium M199. Identical dilutions of the solvent were prepared and used as control. Since nebivolol was previously shown to reduce in a concentration-dependent manner, the intracellular increase of ROS, and superoxide and to reduce them by 50% at 10 *μ*M [3], this concentration was chosen throughout the study and the same time of exposure (24 hours at 37°C) was used.

### 2.2. LDL isolation and oxidation

Whole blood, obtained by venipuncture from healthy volunteers (with informed consent) after 12 hours of fasting, was collected into vacutainer tubes (Becton Dickinson, Meylan, France) containing EDTA (1 mg mL^−1^), and processed for LDL separation in 1 day by sequential flotation in NaBr solution containing 1 mg mL^−1^ EDTA [15]. Cu^2+^ modified LDL (1.7 mg protein mL^−1^) was prepared by exposure of LDL to 5 *μ*M CuSO_4_ for 18 hours at 37°C, and the extent of LDL oxidation was determined by thiobarbituric acid-reactive substances, as previously described [16]. Protein was measured by the Pierce BCA protein assay reagent [13]. Oxidative stress in endothelial cells was induced by the addition of ox-LDL at the concentration of 50 *μ*g protein mL^−1^ as previously reported [16].

### 2.3. Macroarray

Gene activation has been evaluated by cDNA array technology [17] in prepared membranes (SuperArray Bioscience Corporation) with chemiluminescence detection.

The expression of the following genes have been studied.

**Adhesion molecules**

CDH5, ICAM1, ICAM2, ICAM3, ITGA5, ITGAV, ITGB1, ITGB3, OCLN (Occludin), PECAM1 (CD31), SELE (E-selectin), SELL (L-selectin), SELPLG (P-selectin), VCAM1.

**Proteins linked to inflammation**

CCL2 (MCP-1), CCL5 (RANTES), CCL20 (exodus-1), CSF2 (GM-CSF), CSF3 (G-CSF), CX3CL1, IFNB1, IL1B, IL3, IL6, IL7, IL8, IL11, IL14, IL15, TNF.

**Proteins linked to antithrombotic or thrombotic state**

ANXA5 (annexin V), CPB2 (TAFI), F3 (tissue factor), PLAT (tPA), PLAU (u-PA), SERPINE1 (PAI-1), TFPI, TFPI2, THBD (thrombomodulin), VWF.

**Metalloproteinases**

MMP1 (collagenase-1), MMP2 (gelatinase A), MMP3 (stromelysin-1), MMP7 (matrilysin), MMP8 (neutrophil collagenase), MMP9.

**Proteases inhibitors**

TIMP1, TIMP2, TIMP3.

**Endothelins**

EDN1 (ET-1), EDN2 (ET-2), EDN3 (ET-3), EDNRA (ETA), EDNRB (ETB).

### 2.4. Protein measurement

Proteins related to the genes involved in the oxidative stress have been measured in the medium by assay kits based on ELISA system in solid phase. For quantification, the following kits have been used for ICAM-1, E-selectin, P-selectin, IL6, TNF-alpha, MMP-2, MMP-9, TIMP-1, and ET-1 R&D Systems kits (USA); for ICAM-2 and ICAM-3 Bender MedSystems kits (Wien, Austria); for tissue factor, u-PA, PAI-1 American Diagnostica kits (Pfungstadt, Germany).

### 2.5. Statistical analysis

Results are expressed as means ± SD. Statistical analysis was performed by analysis of variance followed by Tukey's test post-hoc analysis. All data were analyzed using the “SPSS 11” program for Macintosh.

## 3. RESULTS

**Figure 1:**
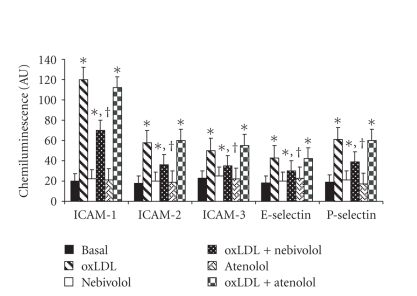
Effect of nebivolol and atenolol on RNA expression of adhesion molecules (ICAM-1, ICAM-2, ICAM-3, E-selectin, and P-selectin) induced by oxidative stress in human umbilical vein endothelial cells (HUVECs). HUVECs were incubated with nebivolol or atenolol (10 *μ*M) for 24 hours at 37°C; oxidative stress was induced by the addition of oxidized (ox)LDL (50 *μ*g protein mL^−1^). *P < .001 versus control; ^†^ *P* < .01 versus ox-LDL. AU = arbitrary units. Data are expressed as means ± SD of 6 different experiments.

Among the genes examined by macroarray, only ICAM-1, ICAM-2, ICAM-3, E-selectin, P ligand-selectin, IL-6, TNF-alpha, tissue Factor, PAI-1, uPA, TIMP-1, MMP-2, MMP-9, and ET-1 were upregulated by the exposure of HUVECs to ox-LDL and, therefore, the protein related to them was measured. The expression of the adhesion molecules studied (ICAM-1, ICAM-2, ICAM-3, E-selectin, P ligand-selectin) significantly increased (*P* < .001) after the exposure of HUVECs to ox-LDL as both RNA (Figure 1) and protein levels (Table 1). Nebivolol, but not atenolol, was able to significantly reduce this increase by 41.7 ± 4.8% for RNA (Figure 1), by 28 ± 3.1% for protein (Table 1) of ICAM-1 (*P* < .01), by 37.9 ± 4.6% for RNA (Figure 1), by 37.6 ± 3.9% for protein (Table 1) of ICAM-2 (*P* < .01), by 30.0 ± 3.5% for RNA (Figure 1), and by 25.7 ± 3.1% for protein of ICAM-3 (*P* < .01), respectively. Similarly, only nebivolol significantly reduced selectins expression induced by ox-LDL by 30.4 ± 3.7% and 36.1 ± 4.1% for RNA (Figure 1) and by 25.1 ± 2.7% and 26.9 ± 3.4% for protein (Table 1), respectively for E-selectin and P-selectin (*P* < .01).

**Figure 2:**
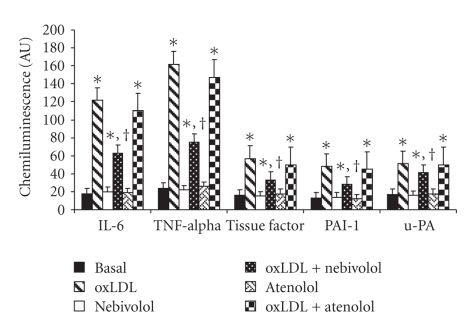
Effect of nebivolol and atenolol on RNA expression of proteins linked to inflammation (IL-6 and TNFalpha) and to thrombotic state (tissue factor, PAI-1, and uPA) induced by oxidative stress in human umbilical vein endothelial cells (HUVECs). HUVECs were incubated with nebivolol or atenolol (10 *μ*M) for 24 hours at 37°C; oxidative stress was induced by the addition of oxidized (ox)LDL (50 *μ*g protein mL^−1^). *P < .001 versus control; ^†^ *P* < .01 versus ox-LDL. AU = arbitrary units. Data are expressed as means ± SD of 6 different experiments.

The expression of both RNA (Figure 2) and protein (Table 1) of molecules involved in inflammation such as IL-6 and TNF-alpha was significantly increased by ox-LDL exposure (*P* < .001). Nebivolol, but not atenolol, was able to reduce this increase by 48.4 ± 5.6% for RNA (Figure 1), by 46.3 ± 4.9% for protein (Table 1) of IL-6 (*P* < .01), by 53.7 ± 5.9% for RNA (Figure 1), and by 41.3 ± 3.9% for protein (Table 1) of TNF-alpha (*P* < .01), respectively.

As shown in Figure 2 and Table 1, the expression of proteins involved in the antithrombotic and thrombotic process such as tissue Factor, PAI-1 and uPA was significantly increased (*P* < .001) by the exposure of HUVECs to ox-LDL. Nebivolol, but not atenolol, at least in part reduced these values as follows: tissue factor −42.1 ± 4.8% for RNA, −30 ± 2.7% for protein (*P* < .01), PAI-1 −41.7 ± 4.6% and −27.1 ± 2.5% for protein (*P* < .01), uPA −35.6 ± 4.1% for RNA, and −25.9 ± 2.8% for protein (*P* < .01).

**Figure 3:**
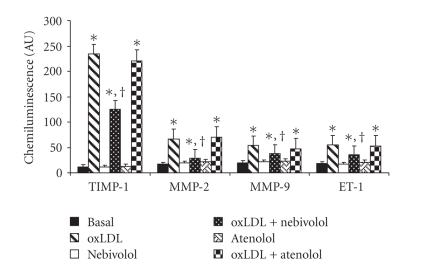
Effect of nebivolol and atenolol on RNA expression of endothelin-1 (ET-1), metalloproteinases (MMP-2, MMP-9) and protease inhibitor (TIMP-1) induced by oxidative stress in human umbilical vein endothelial cells (HUVECs). HUVECs were incubated with nebivolol or atenolol (10 *μ*M) for 24 hours at 37°C; oxidative stress was induced by the addition of oxidized (ox)LDL (50 *μ*g protein mL^−1^). *P < .001 versus control; ^†^ *P* < .01 versus ox-LDL. AU = arbitrary units. Data are expressed as means ± SD of 6 different experiments.

Overexpression of metalloproteinases (MMP-2 and MMP-9) and protease inhibitor TIMP-1 was significantly induced by ox-LDL in HUVECs both as RNA (Figure 3) and protein content (Table 1) (*P* < .001). Nebivolol, but not atenolol, was able to reduce this increase by 56.7 ± 6.3% for RNA (Figure 3), by 43.9 ± 4.1% for protein (Table 1) of MMP-2 (*P* < .01), by 29.6 ± 3.5% for RNA (Figure 3), by 25.5 ± 2.9% for protein (Table 1) of MMP-9, by 46.6 ± 5.4% for RNA (Figure 3), and by 37.7 ± 4.9% (*P* < .05) for protein (Table 1) of TIMP-1 (*P* < .01), respectively. Finally also the expression of ET-1 was significantly increased (*P* < .001) after the exposure of HUVECs to ox-LDL as both RNA (Figure 3) and protein (Table 1). Nebivolol, but not atenolol, was able to reduce this increase by 34.5 ± 3.9% for RNA (Figure 3) and by 40.7 ± 5.7% for protein of ET-1 (Table 1) (*P* < .01), respectively.

## 4. DISCUSSION

In the present study, we have examined the effect of nebivolol and atenolol on gene expression in response to oxidative stress induced by ox-LDL in cultured HUVECs. Ox-LDL is a proatherogenic lipoprotein that has been identified as a potent stimulus for vascular ROS and has been suggested to modulate gene expression of vascular cells [18] by a specific binding to the receptor LOX-1 [16]. The first result of this study is that ox-LDL significantly increased the expression of some genes involved in the formation and progression of atherosclerotic plaque [19]. Our results are in accordance with recent findings showing that ox-LDL upregulates cell-cell interaction, inflammation, growth, apoptosis, and hemostasis genes in smooth muscle cells and macrophages [20, 21].

In this study, we also found that nebivolol decreased ICAMs, P- and E-selectin at the mRNA, and protein level expression in HUVECs exposed to oxidative stress, whereas atenolol was ineffective. Our findings, even in different experimental conditions, agree with a very recent study showing that nebivolol reduced adhesion molecules genes expression in human coronary endothelial and vascular smooth muscle cells [22]. Adhesion molecules mediate the first step in leukocyte extravasation which stands at the very beginning of the cascade of events leading to plaque formation [19]. P- and E-selectins, that are expressed only on activated endothelium, have been demonstrated to play an important role in both early and advanced stages of atherosclerotic lesion development [23]; absence of P-selectin, in fact, has been found to delay fatty streak formation in mice [24]. Several studies have reported increased plasma levels of E-selectin and ICAM-1 in patients with hypertension, diabetes, and hypercholesterolemia [25–27, 34]; moreover in patients at cardiovascular risk, plasma concentration of ICAM-1 can predict adverse outcome [28].

Another important result of this study is the ability of nebivolol, but not of atenolol, to significantly reduce the oxidative stress-induced TNF-alpha, IL-6, and ET-1 gene expression in HUVECs. This finding is in agreement with a previous study showing that nebivolol can reduce ET-1 secretion in human coronary smooth muscle and endothelial cells [29]. Inflammation plays a key role in mediating all stages of atherosclerosis which is considered a chronic inflammatory disease [19]. Proinflammatory cytokines as TNF-alpha and IL-6 have both been shown to induce structural as well as functional alterations in endothelial cells. These cytokines are known to stimulate the production of endothelin and ROS, reduce acetylcholine-induced vasodilatation, and negatively affect the mRNA of endothelial nitric oxide synthase [30].

In our study, nebivolol also significantly reduced the increased gene expression of tissue factor, one of the pivotal thrombogenic stimuli, PAI-1 and uPA induced by ox-LDL. Atenolol on the contrary was ineffective. In this context, it has been recently demonstrated in hypertensive patients that treatment with nebivolol was associated with a favorable modification of hemostatic and fibrinolytic status in addition to its antihypertensive effects [31]. The plasminogen activator (PA) system plays a key role in vascular homeostasis and constitutes a critical response mechanism to cardiovascular injury. It includes the proteolytic activators as u-PA, plasminogen and its degradation product, plasmin, together with the major inhibitors of this system, PAI-1and PAI-2.

An extensive network of additional proteases, inhibitors, receptors, and modulators is directly associated and influenced by the PA system, the largest group being the MMPs and their respective inhibitors TIMPs [32]. MMPs play an important role in atherosclerosis by degrading the extracellular matrix and contributing to weakening of the vascular wall. Moreover, increased expression of TIMP-1 was demonstrated in atherosclerotic plaques of cholesterol-fed rabbit [33].

Our results showing that ox-LDL increased the expression of MMPs in HUVECs confirm other experimental evidences demonstrating that oxidative stress directly activate MMPs posttranslationally [35]. Present findings show for the first time that nebivolol significantly reduced gene expression of MMPs and TIMPs in HUVECs, whereas treatment with atenolol had no effect. This result is likely due to the peculiar antioxidant activity of nebivolol and seems to be independent to its beta-blocker activity since also propranolol was ineffective in reducing MMPs expression in experimental atherosclerosis [36].

In conclusion the results of our study demonstrated that in cultured endothelial cells exposed to oxidative stress nebivolol can reduce the expression of a number of genes involved in the initiation and progression of atherosclerosis. We and others have already demonstrated that nebivolol decreased ROS concentration by affecting the signalling pathways leading to NADPH oxidase activation in endothelial cells [3], in experimental hyperlipidemia [37, 38] and in angiotensin II-treated rats [7]. We can, therefore, speculate that our results depend on the ability of nebivolol to reduce intracellular ROS concentration and hence to restore redox state in endothelial cells.

Our findings suggest that nebivolol, in addition to its antihypertensive effect, can be useful in preventing atherosclerotic complications related to oxidative stress.

## Figures and Tables

**Table 1:** Effect of nebivolol and atenolol on protein expression of adhesion molecules (ICAM-1, ICAM-2, ICAM-3, E-selectin, P-selectin), proteins linked to inflammation (IL-6, TNFalpha), and thrombotic state (tissue factor, uPA, PAI-1), endothelin-1 (ET-1), metalloproteinases (MMP-2, MMP-9), and protease inhibitor (TIMP-1) induced by oxidative stress in endothelial cells.

	oxLDL	Nebivolol	Nebivolol+oxLDL	Atenolol	Atenolol+oxLDL
ICAM-1 (%)	175 + 19*	98 ± 11	126 ± 17*^†^	99 ± 12	162 ± 21*
ICAM-2 (%)	197 ± 28*	95 ± 13	123 ± 25*^†^	98 ± 15	191 ± 32*
ICAM-3 (%)	152 ± 26*	103 ± 16	113 ± 18*^†^	101 ± 16	140 ± 24*
E-selectin (%)	183 + 25*	99 ± 16	137 ± 21*^†^	102 ± 15	180 ± 23*
P-selectin (%)	245 ± 29*	102 ± 14	179 ± 25*^†^	99 ± 17	236 ± 27*
IL-6 (%)	335 ± 39*	98 ± 16	180 ± 21*^†^	97 ± 16	341 ± 37*
TNFalpha (%)	378 ± 46*	100 ± 15	221 ± 31*^†^	102 ± 14	366 ± 48*
TF (%)	196 ± 27*	99 ± 15	149 ± 21*^†^	100 ± 17	199 ± 30*
u-PA (%)	189 ± 24*	99 ± 16	140 ± 25*^†^	98 ± 12	191 ± 27*
PAI-1 (%)	203 ± 30*	101 ± 15	179 ± 25*^†^	98 ± 15	236 ± 27*
ET-1 (%)	275 ± 32*	97 ± 13	163 ± 25*^†^	101 ± 16	266 ± 35*
MMP2 (%)	287 ± 36*	99 ± 13	161 ± 21*^†^	100 ± 15	296 ± 39*
MMP9 (%)	216 ± 29*	101 ± 11	161 ± 19*^†^	99 ± 13	205 ± 27*
TIMP-1 (%)	737 ± 96*	102 ± 13	459 ± 65*^†^	99 ± 12	721 ± 88*

Legend: data are means ± SD and are expressed as % increase versus control; *P < .001 versus control; ^†^ *P* < .001 versus oxLDL.
